# Water Lubrication of Stainless Steel using Reduced Graphene Oxide Coating

**DOI:** 10.1038/srep17034

**Published:** 2015-11-23

**Authors:** Hae-Jin Kim, Dae-Eun Kim

**Affiliations:** 1 School of Mechanical Engineering, Yonsei University, Seoul 120-749, Korea

## Abstract

Lubrication of mechanical systems using water instead of conventional oil lubricants is extremely attractive from the view of resource conservation and environmental protection. However, insufficient film thickness of water due to low viscosity and chemical reaction of water with metallic materials have been a great obstacle in utilization of water as an effective lubricant. Herein, the friction between a 440 C stainless steel (SS) ball and a 440 C stainless steel (SS) plate in water lubrication could be reduced by as much as 6-times by coating the ball with reduced graphene oxide (rGO). The friction coefficient with rGO coated ball in water lubrication was comparable to the value obtained with the uncoated ball in oil lubrication. Moreover, the wear rate of the SS plate slid against the rGO coated ball in water lubrication was 3-times lower than that of the SS plate slid against the uncoated ball in oil lubrication. These results clearly demonstrated that water can be effectively utilized as a lubricant instead of oil to lower the friction and wear of SS components by coating one side with rGO. Implementation of this technology in mechanical systems is expected to aid in significant reduction of environmental pollution caused by the extensive use of oil lubricants.

Water lubrication has been gaining great interest over the recent years with the expectation that it may provide a solution to environmental issues by replacing the use of oil lubricants in various sliding applications^1^,^2^,^3^. The concept of lubricating machines with water has attracted the attention of numerous industries including manufacturing, automotive, ship building, food, and biomedical^1^. The economic impact of realizing water lubrication technology in these industries is tremendous considering the savings that can be derived from conservation of natural resources, reduction of transportation and disposal costs of oil, and performance maximization of mechanical systems in clean environments where oil lubricants cannot be used^1^,^2^,^3^,^4^,^5^,^6^. In this regard, water lubrication can be regarded as a highly desirable green technology that is vital for sustainable future.

Given the enormous impact of water lubrication technology on the environmental and economic issues, the intrinsic properties of water have strongly limited the use of water as a lubricant in practical engineering applications. The limiting factors in using water as a lubricant have been reported in numerous previous works. In particular, it was shown that low viscosity of water resulted in the lubrication film thickness of only ~70 nm which was about 1/100 to 1/1000 of the thickness of conventional oil in hydrodynamic lubrication condition^1^,^5^. Moreover, the use of water as a lubricant in metallic systems caused severe chemical reactions that were detrimental to proper operation of the system^1^. In order to overcome the limitations of water lubrication, various attempts have been made through modification of metallic surfaces with hard coatings that can provide good lubricity in water environment^6^,^7^,^8^. Nitride-based metal coatings such as CrN, CrSiN, TiCN and TiAlN were reported to exhibit relatively low friction and wear in water lubrication conditions^5^,^9^,^10^,^11^. Wang *et al.* demonstrated the friction and wear behaviors of TiN(C) coatings with respect to various carbon content in water^5^. With an optimized content of C in the coating, the friction coefficient of 0.24 and the wear rate of 3.3 × 10^−6^ mm^3^/N·m under water lubrication conditions could be attained. In addition to the nitride-based metal coatings, diamond-like carbon (DLC) has also received much attention as a promising coating material to be used in water lubrication. Ohana *et al.* investigated the tribological properties of DLC film sliding against a Cr-plated ball under water lubrication conditions^6^. Compared to the friction coefficient of ~0.11 obtained by using a steel ball as the counter surface of DLC film, the value could be reduced to ~0.09 by using the Cr-plated ball. It was proposed that Cr promoted the stable formation of water lubricating film on the DLC surface. Furthermore, doping DLC coatings with various materials such as Ti and hydrogen were also suggested to improve the tribological properties of DLC film under water lubrication conditions^4^,^7^. Despite these methods, the use of DLC coatings under water lubrication conditions has a few inherent disadvantages such as brittleness, high residual stress and poor adhesion to the substrate^2^,^4^,^7^.

In addition to utilizing hard coatings in water lubrication, use of additives in water to reduce friction and wear was also proposed. The purpose of using the additives was to overcome the limitations associated with low viscosity of water. Thus, attempts were made to attain sufficient water film thickness by exploiting soluble polymer and biomolecules or insoluble Cu nanoparticles, nanodiamonds (NDs) and graphene as additives^12^,^13^,^14^. Kinoshita *et al.* investigated the effects of using monolayer GO sheets as water-based lubricant additives^15^. With an addition of 0.01 wt.% of GO in water, a low friction coefficient of 0.05 could be maintained for 60000 sliding cycles. Also, Elomaa *et al.* investigated the effects of GO content in water on the friction and wear properties of DLC coatings^16^. Experimental results revealed that the friction coefficient of DLC coating could be significantly decreased to ~0.06 with an addition of 1 wt.% of GO. However, in practical situations, the use of additives in the lubricating fluid may not be a good solution since the additives themselves can be a source of pollution and the cost of the lubricant rise significantly.

Over the past decade, graphene and its derivatives have demonstrated superior friction and wear properties due to their extraordinary intrinsic properties such as high strength and toughness, low shear strength between layers, and ease of functionalization as to lower the interfacial forces^17^,^18^,^19^,^20^,^21^. Recently, a single layer graphene was used as a solid lubricant to achieve low friction and wear in silicon-based NEMS/MEMS applications^22^. Berman *et al.* demonstrated that friction and wear could be reduced by a factor of ~5 with addition of graphene in ethanol^23^. These results indicate the great potential of graphene and its derivatives to be utilized in lubrication applications.

Given the benefits and limitations of water lubrication technology described above, the motivation of this work was to develop a low friction and wear sliding system using pure water as the lubricant. The approach was to exploit the low frictional properties of graphene by coating this material on the surface of the sliding part instead of using it as an additive in water. This concept was verified through assessment of friction and wear properties of rGO coated stainless steel (SS) ball sliding against a stainless steel (SS) plate under water lubrication conditions. The results were compared with those of a bare SS ball sliding against a SS plate in water as well as in oil lubrication. In order to attain sufficient adhesion of the GO coating on the SS ball, electrodynamic spraying process (ESP) was utilized. Regarding the adhesion of the GO coating, Kim *et al.* investigated the frictional behavior of GO coating deposited on a silicon substrate under dry sliding condition^24^. It was demonstrated that though low friction coefficient could be attained, relatively poor adhesion between the GO coating and the silicon substrate was a major concern.

In this work, much effort was devoted to improve the adhesion properties of the GO coating to the substrate. To this end, the substrate was charged during the GO deposition process to improve the affinity between the coating material and the substrate. Also, ESP offered various advantages such as good film uniformity and thickness control, minimization of solution consumption, large area deposition capability, and cost effectiveness^24^. Following the successful deposition of GO coating on the SS ball by using the ESP, the GO coating was fully reduced through hydrazine treatment. A reduction process was employed to overcome the delamination problem of the GO coating during the sliding test. Experiments conducted with the rGO coated SS ball sliding against the SS plate in water lubrication resulted in a stable friction coefficient of ~0.1 for 100000 sliding cycles. The wear rate of the SS plate in water lubrication was measured to be even lower than that of the oil lubrication condition. This remarkable outcome represents the best lubricating performance in water based on graphene coating up to date.

## Results

### Fabrication and characterization of rGO coating on SS ball

Since aqueous solution is utilized in the ESP deposition process, the degree of dispersion of graphene flakes in the solution is an important factor that affects the quality of the coating^24^. Also, since graphene is inherently hydrophobic due to the absence of functional groups on the basal or edge plane, oxidation process is needed to attain hydrophilic properties. As demonstrated by other works, the modified Hummer’s method followed by tip sonication process was carried out to obtain chemically oxidized graphene that is well-dispersed in the aqueous solution^25^. For the solvent, a mixture of EtOH and H_2_O was utilized to enable complete evaporation of the solution during the flight of the droplet from the nozzle to the substrate. Further confirmation of the stability and degree of graphene oxide (GO) dispersion in the aqueous solution was conducted through the zeta potential measurement. The potential values obtained were in the range of −20 to −30 mV which demonstrated that a well dispersed solution of GO was successfully synthesized^26^.

**Figure 1:**
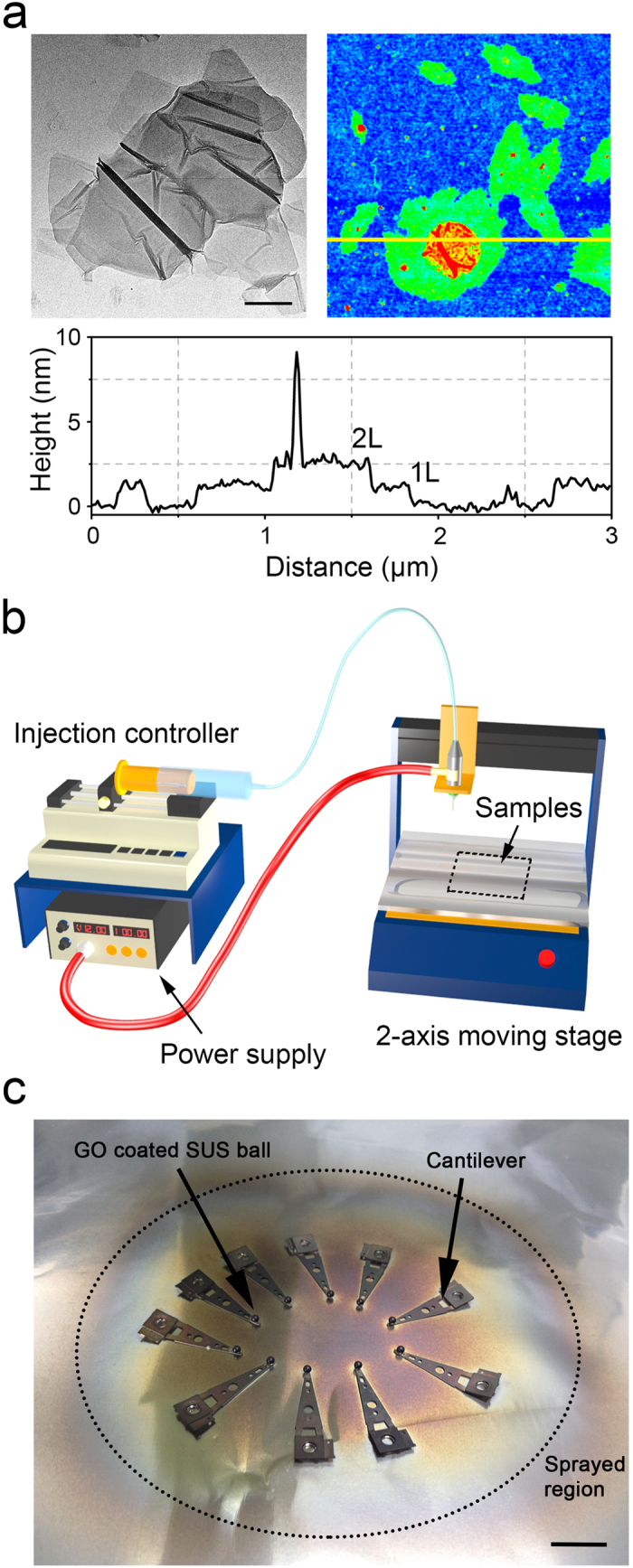
(**a**) TEM (up-left) and 2-D AFM (up-right) images of the graphene oxide sheet. Graph (bottom) is the 2-D surface profile of position marked with yellow line presented in the 2-D AFM image. 1L and 2L in the graph indicate one and two layers of graphene oxide, respectively. Inset scale bar is 200 nm. (**b**) Schematic of the electrodynamic spraying system composed of injection controller, power supply and 2-axis moving stage. Rectangular dotted line indicate the location of the samples during the ESP. The schematic was drawn by H.-J. Kim using conventional 3D modeling software. (**c**) Photograph of multiple samples place on the 2-axis moving stage after the ESP deposition with 100 mL injection volume of GO. Inset scale bar is 1 cm.

To characterize the dimensions of GO particles dispersed in the solution, transmission electron microscope (TEM) and atomic force microscope (AFM) images of the particles were obtained. As shown in the TEM image of a GO particle in the shape of a sheet in Fig. 1a, the size of GO was measured to be ~1 μm. The 2-D surface profile of the position indicated with a yellow line in the 2-D AFM image showed that the thickness of GO single layer was ~1 nm. The height of the region indicated with green and red colors in the 2-D AFM image was ~1 nm and ~2 nm, respectively, which indicated that the single layer GO was partially covered with another layer of GO. Also, it should be noted that the wrinkled morphology of the GO surface shown in the TEM and AFM images is assumed to be due to the water diffusion between GO and the substrate during the preparation of the samples as noted previously^27^.

GO was deposited by ESP on a pre-cleaned 1.6 mm diameter SS ball that was attached to a cantilever specimen holder. The ball was attached to the cantilever using a silver paste to acquire conductivity of the sample which is essential during the ESP. Droplets of the GO solution are deposited on the sample, driven by the electric field applied between the nozzle (+) and the grounded 2-axis moving stage (−) to which the cantilever is mounted. Fig. 1b shows the schematic of the electrodynamic spraying system that is composed of injection controller, power supply and 2-axis moving stage. In the deposition process, multiple samples were placed in the center of the 2-axis moving stage. Fig. 1c shows the photograph of multiple samples after the ESP deposition. The applied voltage to the nozzle was adjusted from 15 to 20 kV until a parabolic shape of the spray could be achieved. The injection speed and volume of GO dispersed solution was set to be 25 μLmin^−1^ and 100 mL, respectively. The thickness of the GO coating on the SS ball was measured to be ~2 μm by using a 3-D laser microscope.

In the preliminary tests, GO was deposited on the SS ball by using the ESP to achieve low friction coefficient under water lubrication condition. However, premature delamination of the GO coating during the sliding tests was found to be the major drawback for GO to be used as an effective lubricant in water lubrication (see Supplementary Fig. S1). Furthermore, despite the effort to control the thickness of the GO coating for prolonged lifetime, the coating was still readily delaminated from the SS ball. It was presumed that relatively high compatibility of GO and water molecules resulted in poor adhesion between the GO coating and the SS ball. Also, it should be mentioned that delamination of the GO coating resulted in the rapid increase in the friction coefficient and high wear rate. Following numerous efforts to increase the durability of the coating, reduction of the GO coating was found to be effective in improving the adhesion of the coating to the SS ball. Thus, the reduction process was an essential fabrication process to overcome the delamination problem of the GO coating during the sliding test under water lubrication condition.

**Figure 2:**
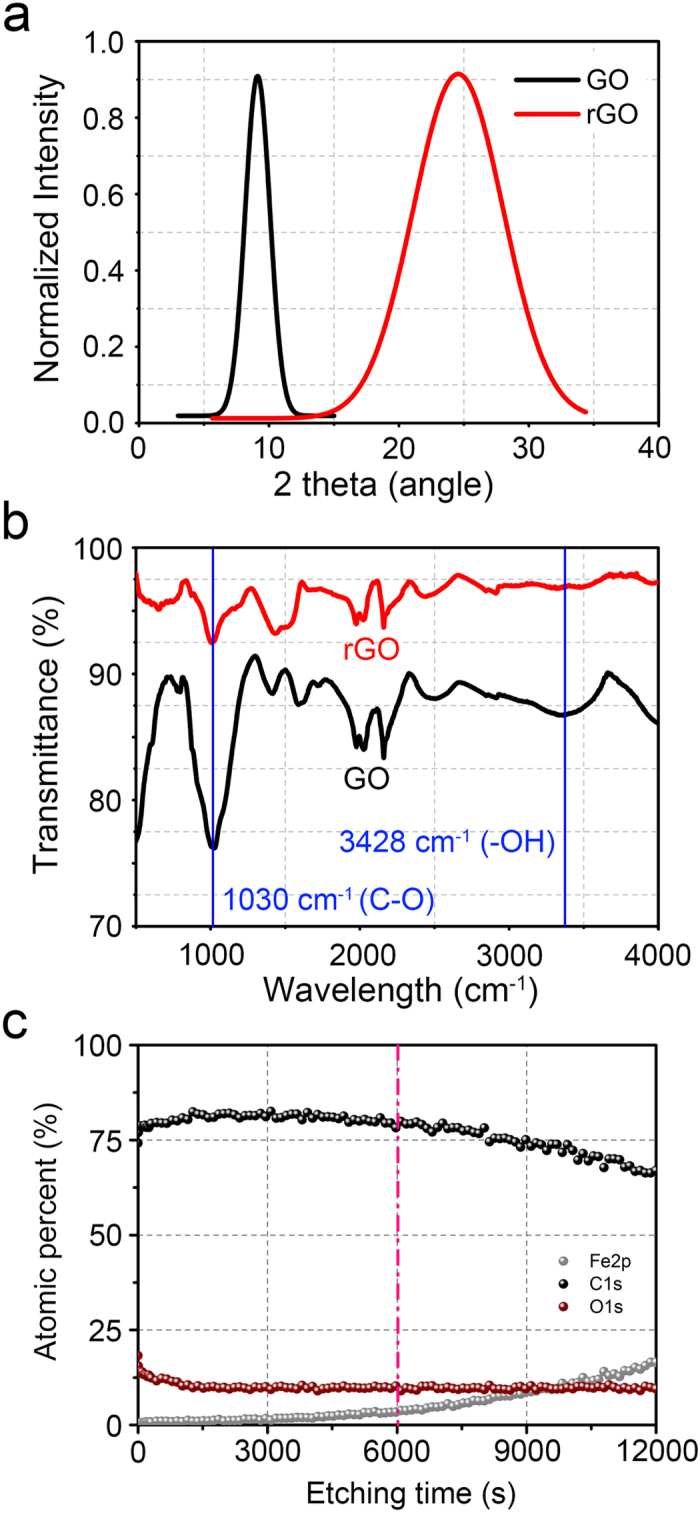
(**a**) XRD pattern of GO (black) and rGO (red). The marked shift of (002) peak from GO (9.2°) to rGO (24.5°) was obtained with reduction process^43^. (b) ATR-FTIR spectra of GO (black) and rGO (red). Blue lines indicate the location of C-O and –OH peaks at the wavelengths of 1030 cm^−1^ and 3428 cm^−1^, respectively. (**c**) XPS depth profiles of the rGO coated SS ball after hydrazine treatment. The pink dotted line indicates the depth at which full penetration of the coating has been reached. This point was determined by noting the beginning of Fe element detection that was associated with the SS ball.

Following the deposition of GO on the SS ball by ESP, reduction process of GO was carried out to obtain reduced GO (rGO). All the samples were placed in an enclosed glass chamber containing vaporized hydrazine for 24 hours at 80 °C. After the hydrazine treatment, all the samples were dried in a vacuum chamber for another 24 hours to remove the hydrazine residue. From the color change of the samples from dark brown to black, it could be preliminarily confirmed that GO was successfully transformed to rGO. Further investigation of the atomic structure and stretching vibration of rGO was performed by using X-ray diffraction (XRD) and attenuated total reflection Fourier transform infrared spectroscopy (ATR-FTIR). Fig. 2a shows the XRD spectrum of GO and rGO with respect to the incident angle (2θ). The (002) plane peak of GO was at 9.2° which corresponded to the interlayer spacing distance of 9.64 Å between the GO layers. After the hydrazine reduction process, the (002) plane peak of rGO was at 24.5° which indicated that the interlayer spacing distance between rGO layers was reduced to 3.62 Å. This value corresponded well with the previously reported value of 3.34  Å for graphite interlayer spacing^28^.

The FTIR spectrum of the GO and rGO samples deposited on the glass substrate are shown in Fig. 2b. The numerous peaks found in the FTIR spectrum indicated that various configurations of carbon and oxygen bonding existed in the GO and rGO. As indicated with the blue line for both GO and rGO at the wavelengths of 1030 cm^−1^ and 3428 cm^−1^, bonding vibrations of carboxyl (COOH) and hydroxyl (C-OH) groups could be obtained. The significant reduction in the peaks of the rGO compared to that of the GO confirmed that the rGO was successfully obtained^29^.

To investigate the chemical composition of the rGO coated SS ball with respect to the depth, the depth profiling XPS analysis was conducted. As the etching rate was unknown for the rGO during the XPS analysis, the detection point of Fe was considered to be the finishing point of the measurement. As shown in the XPS data, the atomic percent values with respect to the etching time of Fe, C and O were obtained. It could be determined from the XPS results that the increase of the Fe atomic percent occurred at ~6000s etching time. Thus, 6000s of etching time was taken as the point to fully penetrate the rGO coating thickness. It was interesting to note that almost no change in the atomic percent for both C and O was found within 6000s. Thus, it was confirmed that the GO coating was fully reduced through its entire thickness after the hydrazine treatment.

### Friction and wear behavior of SS under water and oil lubrication conditions

**Figure 3:**
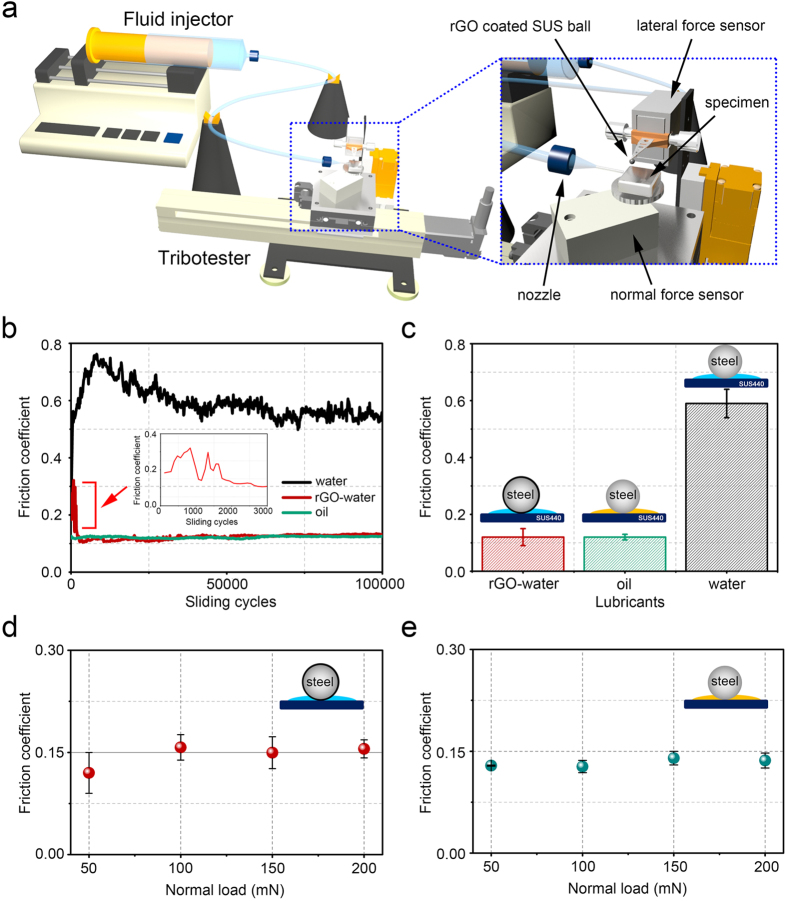
(**a**) Schematic of the reciprocating type of a tribotester with fluid injector attached to provide continuous supply of water or oil lubricant to the sliding system. Schematic indicated with blue dotted line shows the magnified image of the test region. The schematic was drawn by H.-J. Kim using conventional 3D modeling software. (**b**) Friction coefficient under different lubricant conditions with respect to the number of sliding cycles. Inset red graph shows the friction coefficient of rGO-water lubrication condition for the first 3000 cycles. (**c**) Steady-state friction coefficients with respect to different experimental conditions. Each bar is represented by an average value with standard deviation for 3 tests. (**d**) Friction coefficient of rGO-water lubrication condition with respect to different normal loads. (**e**) Friction coefficient of oil lubrication condition with respect to different normal loads. Each data point is represented by an average value with standard deviation of 3 tests.

The friction and wear properties of the rGO coated 440 C SS ball sliding against the 440 C SS plate (10 × 10  mm) in water lubrication was assessed by using a reciprocating type of a tribotester. Water was supplied to the sliding interface continuously at the flow rate of 5 μLmin^−1^ using a fluid injector shown in the schematic of Fig. 3a. During the sliding test, the frictional and normal forces measured by the load cells were monitored in real-time using a data acquisition system. The rGO coated SS ball attached to the cantilever was fixed to the lateral force sensor as shown in the magnified schematic image of the test region indicated with a blue dotted line in Fig. 3a. The normal load was set to be 50 mN which corresponded to a contact pressure of ~0.57 GPa between the SS ball and the SS plate, as calculated by the Hertzian contact equation. The reciprocating speed and stroke were set to be 4 mm/s (1 Hz) and 2 mm, respectively. Sliding tests conducted in both water and oil (mineral oil) lubrication conditions for comparison. Considering the relatively high contact pressure and low sliding speed conditions of the sliding tests, it was presumed that the lubrication state corresponded to that of a boundary lubrication^30^. The sliding tests were performed 3 times for each experimental condition to assure repeatability in a Class 100 clean booth at ambient conditions.

Fig. 3b shows results of the friction coefficient with respect to the number of reciprocating sliding cycles under different lubrication conditions. The water and oil data shown in the graph as black and green lines, respectively, were obtained using the uncoated SS ball sliding against the SS plate. rGO-water data indicated by red line was obtained using the rGO coated SS ball. The tests were conducted for 100000 cycles with a continuous supply of lubricant through the nozzle of the fluid injector. As for the uncoated SS ball sliding against the SS plate under the water lubrication condition, the friction coefficient increased rapidly up to ~0.75 during the first 8000 cycles. The increase in the friction coefficient at the early stage of sliding was attributed to abrasive and corrosive wear of the steel surfaces^31^,^32^. For the next 42000 cycles, the friction coefficient decreased steadily to 0.6. This behavior in the friction coefficient may be due to decrease in the contact pressure as wear progressed to create a larger contact area. Following the decrease of friction coefficient, a steady-state friction coefficient of ~0.55 could be obtained for the remaining sliding cycles. As previously reported in cases where the wear rate of the sample decreased with increasing number of sliding cycles, it may be assumed that the contact pressure has reached the steady state^23^,^31^,^32^,^33^. It should be mentioned that the level of friction coefficient of steel-steel surfaces in water lubrication was quite comparable to the previously reported works^23^,^33^. As for the rGO coated SS ball sliding against the SS plate under the water lubrication condition, a rapid decrease of friction coefficient from ~0.3 to 0.1 occurred for the first 3000 cycles as shown in the inset of Fig. 3b. Following the decrease of friction coefficient during the early stage of sliding, the friction coefficient remained quite steady at ~0.12 for the rest of the sliding cycles. In the case of oil lubrication condition, the friction coefficient was ~0.12 for the entire duration of the sliding test.

Fig. 3c shows the average steady-state friction coefficients with respect to the experimental conditions of the tests that were conducted for 3 times. The average friction coefficients of rGO-water, oil, and water lubrication conditions were 0.12 ± 0.03, 0.12 ± 0.01 and 0.59 ± 0.05, respectively. These results indicated that the combination of rGO coating on the SS ball and water lubrication had comparable frictional properties with that of oil lubrication. Also, compared to the friction between uncoated SS ball and the stainless steel plate in water lubrication condition, the rGO coated SS ball with water lubrication showed 5 times lower frictional force. Thus, the remarkable ability of the rGO coating on SS ball in providing low friction in water lubrication condition that is as low as that of the oil lubrication condition was attained.

**Figure 4:**
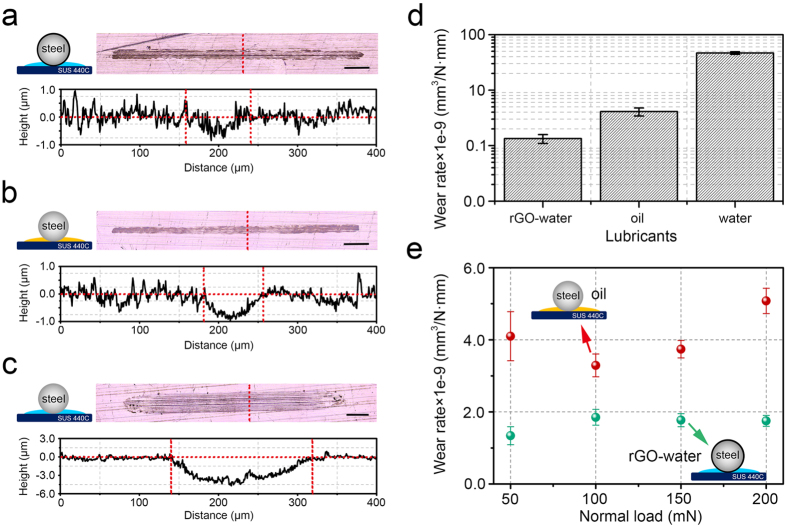
Optical images of wear track (up) and surface profiles (down) of positions marked with red dotted line presented in the optical images after the sliding tests under 50 mN normal load for (**a**) rGO-water (**b**) oil and (**c**) water lubricant conditions. Inset scale bar is 200 μm. (**d**) Wear rate of the steel specimen under various lubrication conditions. Each bar is represented by an average value with standard deviation of 3 tests. (**e**) Wear rate of the steel specimen under water and oil lubrication conditions with respect to different normal loads. Each data point is represented by an average value with standard deviation of 3 tests.

Following the sliding tests conducted for 100000 cycles, the wear tracks generated on the SS plate samples were analyzed using the 3-D laser microscope to quantify the amount of wear. Fig. 4 shows the optical microscope images of wear tracks after the tests and the corresponding 2-D surface profile of the position marked with a red dotted line in the microscope image. The largest amount of wear was found on the SS plate that was slid against the uncoated SS ball in water lubrication.

The amount of wear was quantified from the profiles of the wear tracks formed on the SS plate. The width and depth of the wear track of the SS plate slid against the rGO coated SS ball in water lubrication were measured to be ~80 μm and ~450 nm, respectively. In the case of the sliding test conducted using the uncoated SS ball in oil lubrication, the width and depth of the wear track were measured to be ~73 μm and ~900 nm, respectively. Finally, the width and depth of the wear track was measured to be ~183 μm and ~4.2 μm, respectively, for the case of using the uncoated SS ball in water lubrication. From the 2-D profiles of the wear track, the cross-sectional wear area was automatically calculated using the software provided in the 3-D laser microscope. Then, the wear rate was obtained from the wear volume which was calculated by multiplying the average cross-sectional wear area by the stroke. Fig. 4d shows the wear rate of the SS plate after 100000 sliding cycles for different experimental conditions. The wear rates of the SS plate for rGO-water, oil, and water lubrication conditions were calculated to be 1.34 ± 0.25 × 10^−9^ mm^3^/N·mm, 4.1 ± 0.68 × 10^−9^ mm^3^/N·mm and 46.5 ± 2.54 × 10^−9^ mm^3^/N·mm, respectively. Surprisingly, the wear of the SS plate slid against the rGO coated SS ball in water lubrication was significantly lower than that of the uncoated SS ball in oil lubrication, by a factor of 3. Thus, though the two cases showed similar frictional behavior significantly lower wear of the SS plate could be attained with the rGO coated SS ball.

To investigate the effect of contact pressure on the lubricating performances of the rGO coating, the sliding tests for the rGO-water and oil lubrication conditions under different normal loads were conducted. The applied normal load was set to be 50, 100, 150 and 200 mN which result in a Hertzian contact pressure of 0.57, 0.7, 0.8 and 0.9 GPa, respectively. All other experimental conditions were given the same conditions as previously reported in the manuscript.

Fig. 3d,e show results of the friction coefficient with respect to the normal load under water and oil lubrication conditions, respectively. As for the rGO-water lubrication condition, almost no change in the averaged friction coefficient with increasing normal load was found. The friction coefficient of the sliding tests under 50, 100, 150 and 200 mN normal loads were 0.12 ± 0.03, 0.16 ± 0.02, 0.15 ± 0.02 and 0.16 ± 0.01, respectively. In this regard, it was interesting to note that the lubrication performance of the rGO coating under water lubrication condition could be preserved even at relatively higher contact pressures up to ~0.9 GPa. As for the friction coefficient of the oil lubrication condition, almost constant value of ~0.13 was obtained for all normal loads. The friction coefficient of the sliding tests under 50, 100, 150 and 200 mN normal loads were obtained to be 0.13 ± 0.001, 0.13 ± 0.01, 0.14 ± 0.01 and 0.14 ± 0.01, respectively. Thus, it can be mentioned that the lubricating performance of the rGO coating in water lubrication was comparable to that of the oil lubrication regardless of the contact pressure ranged from 0.57 to 0.9 GPa. Overall, the rGO coatings on SS provided significant friction reduction and superior protection of the SS counter surface under water lubrication conditions.

### Wear characteristics of rGO coated SS ball in water lubrication

**Figure 5:**
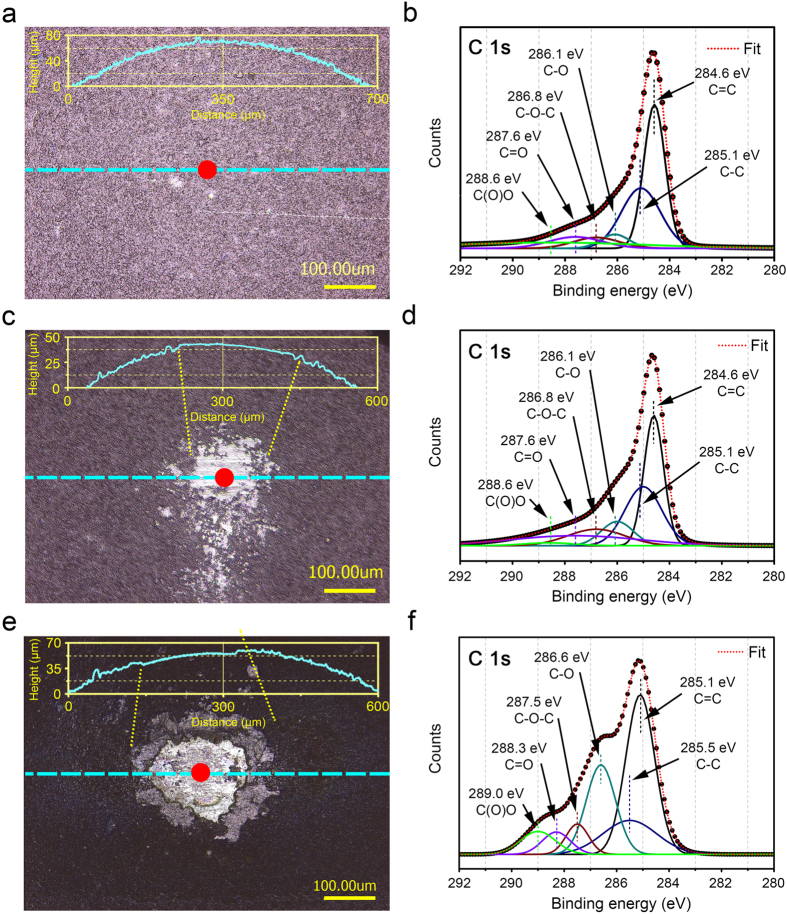
XPS analysis of rGO coated SS ball after the sliding test under water lubrication condition. Optical microscope image (left) and corresponding XPS spectrum of C 1 s (right) of the position marked with a red dot in the optical image of (**a**,**b**) pristine rGO coated SS ball. (**c**,**d**) rGO coated SS ball after 50000 cycles of sliding test. (**e**,**f**) rGO coated SS ball after 100000 cycles of sliding test. Inset graph in optical images represent 2-D surface profile of the position marked with a magenta dotted line for (**a**) pristine rGO coated SS ball (**a**) rGO coated SS ball after 50000 sliding cycles and (**e**) rGO coated SS ball after 100000 sliding cycles.

In order to understand the wear behavior of the rGO coated SS ball sliding against the SS plate in water, X-ray photon spectroscopy (XPS) analysis at the contact area (indicated by a red dot in Fig. 5) on the rGO coated SS ball were performed. Fig. 5a shows the optical microscope image of the pristine rGO coated SS ball before the sliding test. As confirmed by the XRD and FTIR results that showed the successful reduction of GO to form the rGO, the relatively weak peaks of various oxygen configurations in the rGO such as C(O)O, C=O, C-O-C and C-O could be found as shown in Fig. 5b. Relatively strong peaks of C-C and C=C bonds were detected at the binding energies of 285.1 eV and 284.6 eV, respectively. It should be mentioned that the XPS spectra shown in Fig. 5a was in good agreement with the rGO spectrum shown in previous reports^28^,^34^. Fig. 5c,d are the optical microscope images and the corresponding XPS spectra of the rGO coated SS ball after the sliding test conducted for 50000 cycles. As can be noticed from the brighter part of the optical microscope image in Fig. 5c, the asperities of the rGO coating were partially removed which led to generation of a smoother surface. Also, the presence of slight scratches along the horizontal sliding direction on the contact region of the rGO coated SS ball could be found. From the XPS spectra shown in Fig. 5d, it was interesting to note that the peaks of oxygen configurations in the rGO increased. In the meantime, the areal peak ratio of [C-C/C=C] also increased from 0.76 (Fig. 5b) to 0.85 (Fig. 5d). It was presumed that breakage of the C=C bonds led to the increase in the C-O bonds. From the XPS spectra shown in Fig. 5b,d the areal peak ratio of [C-O/C=C] were calculated to be 0.13 and 0.30, respectively. Fig. 5e is the optical microscope image of the rGO coated SS ball that was slid against the SS plate for 100000 cycles. Compared to the case of 50000 sliding cycles, the bright contact region was larger as expected. From the XPS spectra shown in Fig. 5f, it was found that the oxygen peaks increased significantly compared to that of the pristine rGO coating. Considering that the areal ratio of [C-O/C=C] was 0.57, significant oxidation of rGO may have occurred, probably due to the frictional heat generated during the repetitive sliding process. Moreover, the use of water as a lubricant may have accelerated the oxidation process of rGO.

In addition to the XPS analysis, 2-D surface profiles of the SS balls after the sliding tests were obtained by using the 3-D laser microscope as shown in the inset graphs of Fig. 5a,c,e. Compared to the surface profile of the pristine rGO coated SS ball, the surface profile of the ball after 50000 sliding cycles was smoother at the contact region, which indicated that rGO coating was hardly worn during the 50000 sliding cycles. In other word, only asperity level burnishing wear was experienced by the rGO coated SS ball. On the other hand, the 2-D surface profile of the ball after 100000 sliding cycles showed similar smoothening effect of the contact region but with a slightly depressed contour. This suggested that the coating experience some degree of wear. However, a certain amount of rGO coating still remained on the contact region of the SS ball which aided in providing steady and low friction coefficient of ~0.12 up to 100000 sliding cycles.

Following the sliding tests under different normal loads, the degree of wear was quantified by using the 3-D laser microscope. Fig. 4e shows the result of the wear rate with respect to the normal load under water and oil lubrication conditions. As for the oil lubrication condition, the wear rate up to 150 mN load considered to be similar with a value of ~3.7 × 10^–9^ mm^3^/N·mm if the range of the error bars are taken into consideration. However, a significant increase in the wear rate was observed for the 200 mN load condition. In the case of the water lubrication condition, the wear rate was quite steady for loads equal to 100 mN and higher with a value of ~1.7 × 10^–9^ mm^3^/N·mm The lowest wear rate could be achieved under the normal load of 50 mN. Thus, even under varying load conditions, the wear rate of the steel specimen under rGO-water lubrication condition was lower than that of the steel specimen under the oil lubrication condition.

### Mechanism of low friction and low wear of rGO coated SS ball in water lubrication

As described in the sliding test of rGO coated SS ball sliding against the SS plate in water lubrication, the initial friction coefficient was obtained to be ~0.3. The relatively high frictional force may be explained by the poor compatibility between hydrophobic rGO and water. Peng *et al.* assessed the tribological behaviors of rGO and GO layer with respect to the relative humidity (RH%) by using an AFM^35^. As for the rGO single layer, the friction force increased with increasing humidity from 30 RH% to 55 RH%. It was mentioned that the increase in the friction force was a consequence of unstable formation of water meniscus on the hydrophobic surface of rGO. On the contrary, hydrophilic GO showed stable formation of water meniscus on the surface at a relatively high humidity (55 RH%) which enabled effective lubrication of water during the sliding test. In this regard, it may be stated that a relatively high friction coefficient (~0.3) of rGO coating in water lubrication at the early stage of sliding was due to the incompatibility between the water molecules and rGO.

The rapid drop in friction coefficient down to ~0.1 following the initial stage of relatively high friction was attributed to oxidation of rGO. As shown in the XPS spectrum of the rGO coating after 50000 and 100000 sliding cycles, oxidation of rGO coating which led to the formation of GO at the contact region progressed due to repetitive sliding in water under a high contact pressure of ~0.57 GPa between the SS ball and the SS plate. Thus, it was determined that transformation of rGO to GO enabled the stable formation of a lubricating water film during the sliding test.

In addition to the hydrophilic property of GO, the decrease in the interfacial strength within the oxidized rGO (i.e. GO) layers may have also contributed to the significant reduction in friction and wear. Numerous works have been conducted to investigate the mechanical properties of GO and rGO with respect to various conformations of the functional group by using molecular dynamics (MD) simulation^36^. Zhang *et al.* demonstrated that increase in the number of functional groups (epoxy and hydroxyl) in the pristine graphene resulted in the degradation of the interlayer bond strength^37^. It was demonstrated that the elastic modulus of pristine graphene was significantly reduced with respect to the increase in the number of functional groups. In this regard, it may be stated that relatively low interfacial strength within the GO layers was favorable for various functional groups to exist within the layers. This outlook was consistent with the FTIR and XPS spectra shown in Figs. 2b and 5, respectively. Also, increase in the interfacial spacing distance between the GO layers may have increased the possibility of water molecules to be penetrated into the gap. According to previous reports regarding the penetration of the water molecules in between GO layers, one-molecule-thick water layer could be readily placed in between the GO layers with hydrogen bonds^38^,^39^. Thus, it was postulated that the low interfacial strength of GO layers and the water molecules in between the GO layers contributed to lowering the shear strength of the GO coating. Owing to the low shear strength of GO layers during repetitive sliding in water, low friction of 0.1 for 100000 cycles and superior surface protection ability of SS plates could be achieved.

## Conclusions

The concept of lubricating mechanical components using water instead of oil has received great attention in recent years due to its obvious benefits with respect to conservation of natural resources and mitigation of environmental problems. In this regard, numerous studies have been conducted to exploit water as a lubricant by using hard coatings or additives to overcome the inherently poor lubricity of water. Herein, rGO coating was proposed as a method to realize the water lubrication technology for stainless steel materials. A uniform GO coating was successfully deposited on a SS ball which was used to slide against a SS plate in water and oil lubrication conditions. The GO coating could be reduced to form rGO by the hydrazine reduction process. The friction and wear behavior of the rGO coated SS ball was assessed under different sliding conditions. The friction coefficient of the rGO coated SS ball sliding against the SS plate in water lubrication was ~0.12 over 100000 sliding cycles. This value was significantly lower than the value of ~0.6 obtained by using the uncoated SS ball in water lubrication and similar to the value obtained in oil lubrication using the uncoated SS ball. Following the sliding test, the wear track was analyzed with a 3-D laser microscope to quantify the wear rate. It was revealed that wear of the SS plate slid against the rGO coated SS ball in water lubrication was significantly lower than that of the uncoated SS ball in oil lubrication, by a factor of 3.

The mechanism of low friction and wear characteristics of the rGO coating in water lubrication was analyzed. XPS results showed that rGO was oxidized during the sliding test under the water lubrication condition. It was confirmed from the XPS spectra that the C-O peak intensity increased with respect to the increase in sliding cycles from 50000 to 100000, which indicated that rGO transformed to GO. Due to the increased interlayer distance and hydrophilic property of GO, the penetration of water molecules in between the GO layers could be achieved, which resulted in low shear strength of the layers^36^,^37^. Also, it was found that improved lubricity of rGO under water lubrication condition aided in lowering the wear rate of the SS plate.

Considering the superior lubricating properties of the rGO coating under water lubrication condition, the influence of water molecules on the friction and wear reduction effect was considered to be quite significant. It was postulated that penetration of the water molecules in between the rGO layers may have resulted in lowering the shear strength of the rGO layers. Numerous works regarding the penetration of water molecules in between the GO layers have been conducted with the aim to comprehend the underlying mechanism of this phenomenon. Nair *et al.* have conducted an interesting experiment showing that the evaporation rate of water molecules through an open aperture was quite comparable to that of water molecules evaporated through the aperture covered with GO^40^. This outcome suggested that the water molecules were highly permeable through the GO layers. To verify the mechanism of water permeation through GO, molecular dynamics (MD) simulation was employed. It was revealed from the MD simulation results that the oxidized part of graphene played an important role in creating a space between the graphene layers up to 7 ~ 10 Å. It should be mentioned that this outcome was in good agreement with the AFM results which showed that the thickness of the GO layer was ~1 nm (Fig. 1a). Also, it was reported that the hydrogen bonding between the epoxy and the hydroxyl groups in graphene and water molecules enabled the water molecules to be present within the GO layers. Furthermore, Rezania *et al.* investigated the change in the interlayer distance of bi-layered GO layers by using the scanning force microscopy (SFM) to demonstrate the hydration of bi-layered GO layers with respect to different humidity levels as well as in water^41^. It was revealed from the experiment that continuous expansion of the GO interlayer distance occurred at humidity levels below ~80%. When the GO layers were immersed in water, a monolayer of water molecules could be inserted between the hydrated GO layers leading to further increase in the interlayer distance of 3 Å (total interlayer GO distance up to ~12 Å). Thus, it can be stated that the oxidized part of graphene layers resulted in the ease of penetration of the water molecules in between the GO layers.

In regard to the reduction of shear strength of GO layers due to the penetration of the water molecules in between the GO layers, it can be explained with the permeation process of the water molecules in between the GO layers as demonstrated in a previous work. Raghav *et al.* investigated the mobility of the water molecules that were initially present in between the GO layers by using the potential mean force (PMF) calculation^42^. It was found from the calculation that the water molecules first penetrated in between the GO layers due to the presence of the epoxy and hydroxyl groups in graphene. With continuous supply of water molecules, the water molecules accumulated in the GO layers. This accumulation of water molecules in the oxidized part of graphene aided in swelling of the GO layers. Following the swelling of GO layers, the GO layers separated due to the large internal force exerted on the GO layers. Considering the fact that the GO layers were entirely immersed in water during the tests in the present work, the separation of the GO layers due to the swelling of the GO layers could also be expected. As a consequence, the relatively low friction coefficient obtained in this work was attributed to the reduction of shear strength of the GO layers due to penetration of water molecules.

The overall friction and wear performance of the rGO coating in water lubrication was compared with previous works on water lubrication techniques using graphene-based materials. The results of these works were compared with respect to lubricant type, total sliding distance, friction coefficient, and wear amount. As can be confirmed from Table 1 that summarizes the main results of the other works on graphene-based water lubrication techniques, friction coefficient in the range from 0.05 to 0.2 could be achieved by using the graphene-based materials as additives in pure water. When the graphene-based material was used as a coating as demonstrated in this work, adequate lubricating performance with a friction coefficient of ~0.1 could be achieved. As for comparison of the wear amount, either the wear scar diameter or the wear rate was used, depending on the information that was available from the reference source. As shown in Table 1, the wear amount achieved in this work was the lowest among all the previous works. Considering the excellent wear resistance and low frictional behavior of rGO coated stainless steel in water lubrication, the realization of practical water lubrication technology for stainless steel materials looks promising. The development of the rGO coatings for water lubrication applications is expected aid in performance maximization of various mechanical systems with enormous economical benefits.

## Experimental Procedure

Graphene oxide (GO) sheets were synthesized from graphite flakes (Sigma-Aldrich) of 20 μm in size by using the modified Hummer’s method. After several repetitions of tip-sonication process, well dispersed GO sheets in water was prepared. Exfoliated GO sheets were characterized by using a transmission electron microscope (TEM, JEOL-2010) and an atomic force microscope (AFM, Park systems NX10). The interlayer distance between the layers of GO and rGO was confirmed by using an XRD equipped with a Cu_kα_ radiation source (Rigaku-G2005304). The concentration of the GO dispersed solution was 0.2 mgmL^−1^. The stability of the solution was characterized by using a zeta-potential analyzer (Otsuka electronics, ELSZ-1000). During the ESP, the solution was injected through a nozzle at a flow rate of 25 μLmin^−1^ with the total volume of 100 mL. The applied voltage to the nozzle was set in the range of 15 to 20 kV until a stable parabolic shape of the spray could be achieved. The hydrazine reduction process was conducted by placing GO coated SS ball in the enclosed chamber with vaporized hydrazine (N_2_H_4_ 64–65%, reagent grade, 98%). The enclosed chamber was placed on the hot-plate at 150 °C for 24 hours. Following the GO reduction process, the coated SS samples were completely dried in the fume hood for another 24 hours.

Sliding tests were conducted using a reciprocating type of a tribotester with a sliding speed and stroke of 4 mm/s and 2 mm, respectively. The sliding speed was considered to be constant within a single stroke followed by the rapid reversal in the sliding direction at both ends of the stroke. The applied normal load was set to be 50, 100, 150 and 200 mN which result in a Hertzian contact pressure of 0.57, 0.7, 0.8 and 0.9 GPa, respectively. The friction and normal forces were measured by using force sensors in both directions (Transduced Techniques, GS0-10). The data acquisition rate was set to be 10 Hz. The observation of the wear track was performed by using a 3-D laser microscope (Keyence, VK-2000) equipped with the software that can automatically calculate the cross-sectional wear area. The elemental compositions were analyzed by using an XPS equipped with mono-chromated AI Kα (Thermo UK).

## Additional Information

**How to cite this article**: Kim, H.-J. and Kim, D.-E. Water Lubrication of Stainless Steel using Reduced Graphene Oxide Coating. *Sci. Rep.* **5**, 17034; doi: 10.1038/srep17034 (2015).

## Supplementary Material

Supplementary Information

## Figures and Tables

**Table 1:** Recent works on the graphene-based water lubrication techniques.

No.	Lubricant type	Total sliding distance	Friction coefficient	Wear quantification	
				Wear scar diameter	Wear rate (mm^3^/N·mm)
Ref [15]	GO additives	300 m	~0.05	Marginal (No numbers indicated)	
Ref [16]	GO additives	36 m	~0.06	–	7.5 × 10^−9^
Ref [17]	GO additives	1.9 m	~0.1	230 μm	–
Ref [33]	GO additives	18.8 m	~0.2	220 μm	–
Present work	rGO coating	400 m	~0.1	70 μm	1.34 × 10^−9^
