# Supplementary material for: Water Lubrication of Stainless Steel using Reduced Graphene Oxide Coating
Source: Sci Rep. 2015 Nov 23;5:17034. doi: 10.1038/srep17034 (PMC4655472; doi:10.1038/srep17034)
Supplement: Supplementary Information [file srep17034-s1.docx]

**Supplementary Information**

**Water Lubrication of Stainless Steel using Reduced Graphene Oxide Coating**

Hae-Jin Kim^1^ and Dae-Eun Kim^1^

^1^School of Mechanical Engineering, Yonsei University, Seoul 120-749, Korea

**1. Water lubrication of stainless steel by using graphene oxide (GO) coating**

In the preliminary tests, GO was deposited on the SS ball by using the electrodynamic spraying process (ESP) to achieve low friction coefficient under water lubrication condition. The Fig. S1 shows the friction coefficient obtained by using the GO coated ball that was slid against the SS plate in water lubrication. It was found that a very low friction coefficient of ~0.02 could be achieved for the certain sliding distance. Nevertheless, the short lifetime was considered to be the major drawback in using the GO coating as a lubricant in the water lubrication technique.


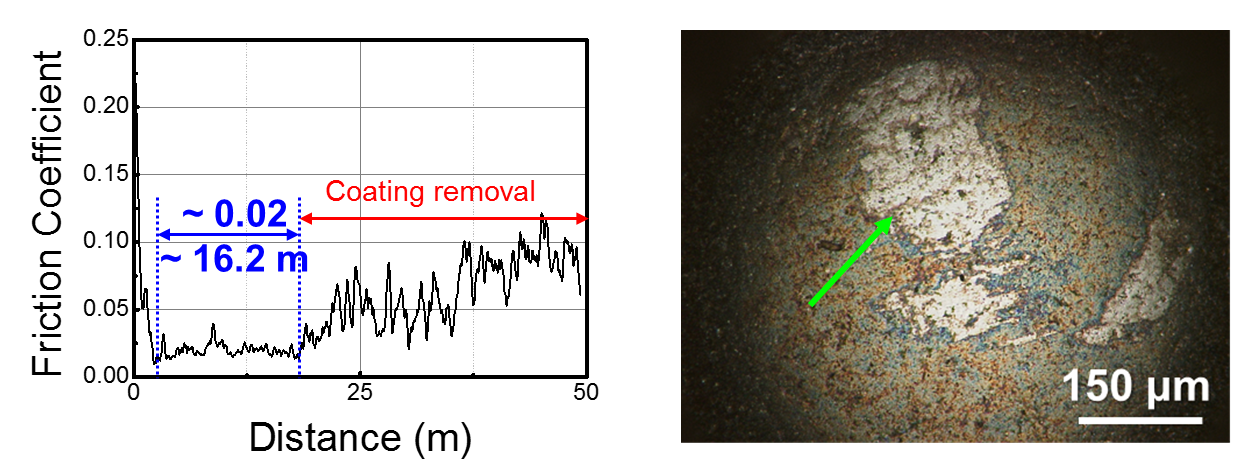


**Figure S1.** Friction coefficient of GO-water lubrication condition with respect to the number of sliding cycles (left) and optical microscope image of GO coated SS ball after the sliding test under water lubrication condition. Green arrow indicates the delaminated region of GO coating after the sliding test.

Furthermore, despite the effort to control the thickness of the GO coating for prolonged lifetime, the coating was still easily delaminated from the SS ball during the sliding test in water lubrication. It was presumed that relatively high compatibility of GO and water molecules resulted in poor adhesion between the GO coating and the SS ball. Also, it should be mentioned that the delamination of the GO coating during the sliding tests resulted in rapid increase in the friction coefficient and high wear rate. To increase the durability of the coating, extensive effort was made, and as a result, reduction of the GO coating was found to be effective in improving the adhesion of the coating to the SS ball. Thus, the reduction process was an essential fabrication process to overcome the delamination problem of the GONS coating during the sliding test under water lubrication condition.
